# Yoga, Meditation, Breathing Exercises, and Inflammatory Biomarkers with Possible Implications in COVID-19: A Systematic Review and Meta-Analysis of Randomized Controlled Trials

**DOI:** 10.1155/2022/3523432

**Published:** 2022-10-07

**Authors:** Komal Shah, Chiranjivi Adhikari, Shubham Sharma, Somen Saha, Deepak Saxena

**Affiliations:** ^1^Indian Institute of Public Health Gandhinagar (IIPHG), Gandhinagar 382042, India; ^2^Pokhara University, School of Health and Allied Sciences, Pokhara 30, Kaski 33700, Nepal; ^3^Datta Meghe Institute of Medical Sciences (DMIMS), Wardha 442107, Maharashtra, India

## Abstract

**Introduction:**

COVID-19, a multisystem disease, has implications for various immunity and infection biomarkers. Yoga (Y), meditation (M), and pranayama (P), and their combinations have shown positive changes on those biomarkers among other than COVID-19 patients and healthy people. So, we aimed to document the evidence of possible implication in a systematic way.

**Materials and Methods:**

We screened 84 full texts, published in the last ten years, from three databases, from which only 44 met the eligibility criteria, and then extracted the data related to demographic characteristics, intervention, results, and strengths and limitations in two MS-Excel grids, and then presented them in tables and figures. Furthermore, we carried out meta-analysis including subgroup and sensitivity analysis using a random effects model of 11 RCTs and reported the mean difference, heterogeneity, and *p* value with 95% CI and presented them with forest and funnel plots and the tables.

**Results:**

Twenty-five biomarkers of 4023 participants (range, 15–413) from 13 countries, healthy and clinical, from both sexes above 18 years, and from mainly clinical settings, were reported. YMP intervention, in solitary or in different possible combinations with varied durations among clinical and pregnant (range, 960–4800 minutes) and healthy (960–8400 minutes, excluding two studies of 20 minutes only) participants, was reported. It was revealed that 25 biomarkers, nine among the apparently healthy, 14 among the patients, and two among the pregnant, changed favourably (*p* < 0.05). Furthermore, either in meta- or subgroup-analysis, mean differences of IL-6 (−1.44 pg/ml) (95% CI) (−2.33, −0.55), (*p* = 0.002, *I*^*2*^ = 82%), Cortisol (−40.75 pg/ml) (95% CI) (−64.13, −17.38), (*p* = 0.0006, *I*^*2*^ = 87%), and TNF-*α* (−3.40 pg/ml) (95% CI) (−4.83, −1.98), (*p* < 0.0001, *I*^*2*^ = 79%) showed statistically significant changes. Nonetheless, considerable heterogeneity and publication bias were observed among the studies.

**Conclusion:**

Although more than two dozens of biomarkers in individual studies showed favourable changes, only IL-6, Cortisol, and TNF-*α* produced significant combined results, even then with much less certainty. Further meta-analysis of biomarkers of COVID-19 patients is highly recommended. Registration: CRD42021283894.

## 1. Introduction

Yoga, meditation, and pranayama, or yogic breathing, have been practised worldwide since antiquity. Research, both here and elsewhere, has shown that practises like guided asanas, specific pranayama, and meditation can improve the body's immune system responses. To develop a holistic framework for individuals' health, the interrelationships between mind and body have been extensively studied in recent times [1–5]. With the advent of the pandemic, researchers and clinicians are in search of practises and therapeutics that can reduce the impact of COVID-19 on human health. In this line, recent reviews of complementary and alternative medicine (CAM) and traditional Chinese medicine (TCM) for COVID-19 have provided preliminary evidence of effectiveness [6, 7]. In addition, a recent review also underscored that the effects of natural compounds from Nigella sativa are promising [8]. It has been stated that as a result of COVID-19 disease, leukocytes, neutrophils, cytokine levels [IL-2R, IL-6, IL-8, and IL-10], and infection biomarkers like CRP, PCT, and ferritin are significantly increased [9, 10]. Multiple studies have shown that yoga, meditation, and pranayama interventions are efficacious in regulating an array of biomarkers, including cytokines [4, 5].

Further research in this area is ongoing since the disease is still uncertain in many aspects, especially regarding its long-term complications [10] and the outbreak of possible new mutants due to natural selection [11]. During the cytokine storm that occurs in COVID-19 syndrome, proinflammatory cytokines are released uncontrollably. A positive relationship between the severity of the viral infection and the mortality rate was observed following an uncontrolled and dysregulated secretion of inflammatory and proinflammatory cytokines [12]. Recent evidence also shows that systemic vasculitis and cytokine-mediated coagulation disorders act as the principal actors of multiorgan failure among COVID-19 patients with severe complications [10]. Assessing the effect of different biomarkers in COVID-19 can help in the early diagnosis of the disease, confirming and classifying the disease severity, identifying the high-risk cohort, framing intensive care unit (ICU) admission criteria, rationalising therapy, assessing response to therapies, predicting outcomes, and framing criteria for discharge from the ICU and/or the hospital [9]. So, it is the need of the hour to assess and identify measures that can control the dysregulated secretion of cytokines and other inflammatory mediators with possible implications for COVID-19. Till date, multiple studies have shown that yoga, meditation, and pranayama interventions are efficacious in regulating an array of biomarkers, including cytokines [4,5]. However, these interventions and their outcomes presented over different settings and durations have shown mixed results. Considering the inconclusive evidence present, the heterogeneity in outcomes, and the duration of interventions given in the previous studies, we sought to systematically review the evidence of the effects of yoga, meditation, and pranayama interventions on COVID-19 related inflammatory biomarkers by including only randomized controlled trials (RCTs).

## 2. Materials and Methods

We conducted this review according to the Preferred Reporting Items for Systematic Reviews and Meta-Analyses (PRISMA) guidelines.

### 2.1. Study Selection Criteria

A systematic literature search was carried out after registering the review protocol in the international prospective register of systematic reviews (PROSPERO Regd. No. CRD42021283894). Peer-reviewed RCTs published during the last 10 years (2010–2021) were included. We sought to identify studies that used any version of yoga, meditation, and/or pranayama, intervened as solitary or in combination, among healthy and clinically ill individuals in adults (≥18 years), including pregnant women, to assess the effectiveness of biomarkers related to or influenced by COVID-19. We were also guided by our earlier scoping review on yoga, immunity, and COVID-19 for study selection [13]. The search was conducted mainly in three databases: The Cochrane Library, PubMed, and Google Scholar. Additionally, we also carried out a citation search. Keywords and MeSH terms were informed by previous literature searches. Six sets of search terms were used including terms related to (i) yoga, (ii) meditation, (iii) pranayama, (iv) intervention, (v) biomarker, and (vi) controlled trial (S1_ Search Strategy with Keywords and MeSH).

### 2.2. Data Extraction and Analysis

Based on our previous scoping review of yoga, immunity, and COVID-19 [13], we developed and then extracted data using two standardized MS-Excel grids. The data extraction form were initially piloted (*n* = 2 studies) and then refined and finalised by all reviewers. The data were extracted by two reviewers (CA and ShS), and the third reviewer (KS) assessed when there was a discrepancy. Pre-post effectiveness of YMP on biomarkers was found using mean difference, difference in differences of means (∆1-∆2), and effect sizes (Cohen's d). However, we only reported *p* values along with the direction of effectiveness in interpreting the tests. The basic characteristics and detailed findings of the included studies are given in Tables 1 and 2, respectively.

For data synthesis, we developed an Excel grid for the main outcomes (biomarkers). The evidence was synthesised into a narrative form. A descriptive analysis of the characteristics of study populations was carried out using range values and proportions.

For meta-analysis, we calculated the mean difference when at least three studies consistently reported the primary biomarkers. We produced forest plots, funnel plots, and risk of bias (ROB) assessment charts using the Cochrane risk of bias tool, from RevMan 5.4.1. The effect size of the meta-analysis was estimated through random effects, considering the post-pre mean change in biomarker concentration and standard deviation (SD). Studies that reported standard mean error (SME) were converted to SD, and for those not specifying SME or SD, either of the two was considered. Following the Cochrane guideline, we calculated the pooled SD from the given parameters, including an additional value of the correlation coefficient, assuming 0.8. Regarding three-armed studies, we pooled the mean and SD from the two experimental groups for the outcome measure.

Subgroup analysis was performed post hoc by stratifying the studies based on intervention type such as yoga, meditation, pranayama, or yoga only, or meditation only, and intervention duration in weeks and minutes. For this, we divided IL-6 into six subgroups, cortisol into two, and TNF-a into three.

## 3. Results

### 3.1. Search Result

**Figure 1:**
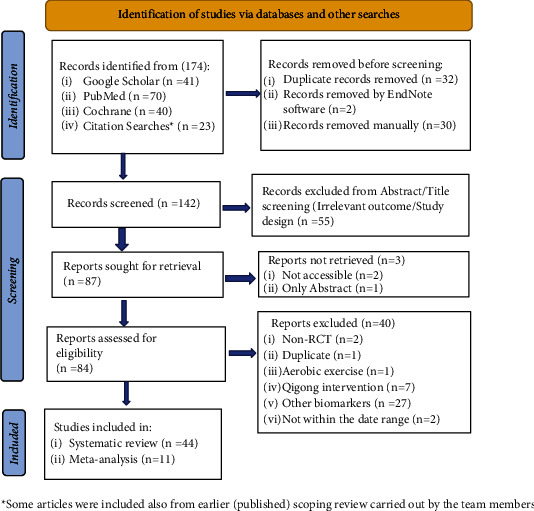
PRISMA flow chart and screening of studies.

An initial search yielded 174 articles. After removing the duplicates and excluding the articles assessing the title and abstract, 87 articles were retrieved for full text. We removed 40 full-text articles that failed to meet the inclusion criteria, as well as 3 articles that were unavailable in full text. Finally, 44 articles were selected for the review (Figure 1).

### 3.2. Description of Included Studies

The study consisted of randomized control trials ranging from less than one week (including two studies which had an intervention of 20 minutes) to 28 weeks (including one study which had 12 months of intervention). The majority of the interventions ranged from 5 to 16 weeks (*n* = 35) [14–48], either solo (Y or M or P) or in combinations (YM, YP, MP, and YMP). There were studies providing yoga (*n* = 2) (Y) [29, 44], pranayama (*n* = 2) (P) [3, 28], meditation (*M*) (*n* = 11) [14–20, 49–52], meditation and pranayama (*n* = 2) (MP) [21, 53], yoga and meditation (*n* = 4) (YM) [32, 33, 35, 36], yoga and pranayama (*n* = 8) (YP) [22–24, 27, 30, 31, 54, 55], and yoga, meditation, and pranayama (*n* = 15) (YMP) [25, 26, 34, 37– 43,45–48, 56] as interventions. Most of the studies had two groups (intervention and control), while 6 studies were reported to have 3-experimental arms. The studies were carried out in 13 countries, with the maximum number of studies coming from the USA (*n* = 19, 43%), followed by India (*n* = 13, 30%), Iran (*n* = 2, 5%), and one each in China, Thailand, Spain, Netherlands, Sweden, Republic of Korea, Brazil, Australia, Taiwan, and Portugal. The interventions given included Iyengar, Patanjali Raj, Hatha yoga, and other forms of yoga, mindfulness-based stress reduction (MBSR), and pranayama (Tables 1 and 2).

### 3.3. Study Quality

Of the 44, twenty-three RCTs were open-label, followed by 19 single-blinded and 2 double-blinded trials. Among the 37 full RCTs, one was a crossover design. The comparator arms included 23 controls or wait-list or attention or observational controls, 11 usual or standard care, and 10 were given placebo or active control. Placebo interventions included health education (HE) with or without supportive therapy, counselling, exercise, rehabilitation, relaxation, healthy living workshop (HLW), dietary intervention, physical exercise, sleep hygiene education (SHE), and stress reduction. Risk of bias (ROB) of 11 studies included in meta-analyses, three were found to be low, one medium, and seven with high risk (Table 3).

### 3.4. Participants

A total of 4023 participants were included in 44 trials, ranging from 15 to 413 participants. Most participants were patients, including those either from in-patient or out-patient clinics or hospital settings. Only a few studies (*n* = 4) were carried out in community and educational institutions. Almost all studies included both men and women aged 18 years or above, whereas some trials were exclusively conducted only on females (*n* = 13) [14, 20, 26, 27, 29–31, 46, 48, 51, 52, 54, 56] (Tables 1 and 2).

### 3.5. Intervention

The trials encompass various forms of asanas, deep relaxation, and other techniques of yoga, meditation, and pranayama. The participants (intervention group only) learned the poses and techniques in the presence of an instructor, and then they were asked to practise the learning at home or in their free time. In only one study, participants learned through an online platform. Except for two studies [16, 19], the daily duration of intervention was less than an hour (*n* = 10) [3, 18, 21–23, 25, 37, 41, 53], one to two hours (*n* = 25) [14, 20, 24, 26–32, 35, 38–40, 42–49, 52, 55] and more than two hours. The intervention duration of the trial by Banasik and colleagues [31] was taken from the authors' previous study [57] (*n* = 7) [15–17, 33, 34, 36, 50].

### 3.6. Outcomes

#### 3.6.1. Yoga (Y)

Two studies that included only yoga as an intervention reported soluble tumour necrosis factor receptor II (sTNF RII) (a cell surface receptor for the proinflammatory cytokine), C-reactive protein (CRP), interleukin 1 receptor antagonist (IL-1RA) [29], interleukin 2 (IL-2) [44], cortisol [29], and Interleukin 6 (IL-6) [29, 44]. The duration of intervention ranged between 1440 and 2160 minutes.

#### 3.6.2. Pranayama (P)

Two studies [3, 28] used pranayama as an intervention with a duration ranging from 20 to 720 minutes. The following biomarkers were studied: IL-6, CRP, 6 minute walk distance (6MWD), diffusing capacity of the lungs for carbon monoxide (DLCO), forced expiratory volume in 1 sec (FEV1), inspiratory capacity (IC), residual volume to total lung capacity ratio (RV/TLC), alveolar volume to total lung capacity ratio (VA/TLC), and inspiratory time to total breathing cycle time ratio (Ti/Ttot) [28]. A second study reported monocyte chemoattractant protein 1 (MCP-1), interleukin 8 (IL-8), interleukin 1 beta (IL-1*β*), IL-1RA, IL-6, interleukin 10 (IL-10), interleukin 17 (IL-17), interferon*γ* induced protein-10 (IP-10), macrophage inflammatory protein 1-beta (MIP-1b), and tumor necrosis factor (TNF-*α*) [3].

#### 3.6.3. Meditation (M)

A total of 11 studies [14–20, 49–52] had only meditation as the intervention. The duration of the intervention ranged from 20 minutes to 12 months. The following biomarkers were reported in the trial: CRP [14, 15, 49], IL-1 beta, TNF-*α* [49], interferon gamma (IFN- *γ*) [16, 49], IL-8 [17], sTNFR II [14], cortisol [18–20], IL-6 [14, 15, 18, 49–52], immunoglobulin A (IgA) [16], IL-10 [16, 50–52], high-sensitive C-reactive protein (hsCRP) [50–52], and IP-10 [15].

#### 3.6.4. Meditation and Pranayama (MP)

Two studies had a combination of meditation and pranayama [21, 53], ranging from a duration of 336 to 1200 minutes. IL-6, TNF-*α* [53], and hsCRP [21] were studied in these trials.

#### 3.6.5. Yoga and Meditation (YM)

With four studies having both yoga and meditation [32, 33, 35, 36] intervention, ranging over a duration of 720 to 1740 minutes, including both learning and practising. Biomarkers included in the study were hsCRP [32, 33], IL-6 [32, 33, 35], extracellular superoxide dismutase (EC-SOD/SOD) [32], CD4+ [33, 36], and TNF-*α* [35].

#### 3.6.6. Yoga and Pranayama (YP)

Eight studies were spanned from 1 hour to 28 weeks of yoga and pranayama intervention [22–24, 27, 30,31, 54, 55]. The following markers were studied: IL-6 [23, 24, 27], hsCRP [22–24], cortisol [22, 30, 31, 54], TNF-*α* [23, 24, 27, 55], TBARS and EC-SOD/SOD [23], IL-1 beta [27], IL-8, IL-10, IL-2, IL-12, IFN-*γ*, IL-4, tidal volume (TV), forced vital capacity (FVC), and FEV1 and peak expiratory force (PEF) [55].

#### 3.6.7. Yoga, Meditation, and Pranayama (YMP)

A total of fifteen studies had a combination of three forms of intervention [25, 26, 34, 37–43, 45–48, 56]. The following biomarkers were found studied in the trial: IL-1*β* and IL-10 [37], IL-1*β* [26], CRP [38–41], IL-17A [40, 42], IL-6 levels [25, 26, 39–41, 43, 45, 46], TNF-*α* [25, 26, 39, 41, 42, 45, 47], cortisol [25, 47, 48, 56], TBARS, SOD [45, 47], IFN-*γ* [47], hsCRP [34, 43, 46], IgA [56], IL-1*α* [25], IL-8, and MCP-1 [26] (Tables 1 and 2).

#### 3.6.8. Meta-Analysis

**Figure 2:**
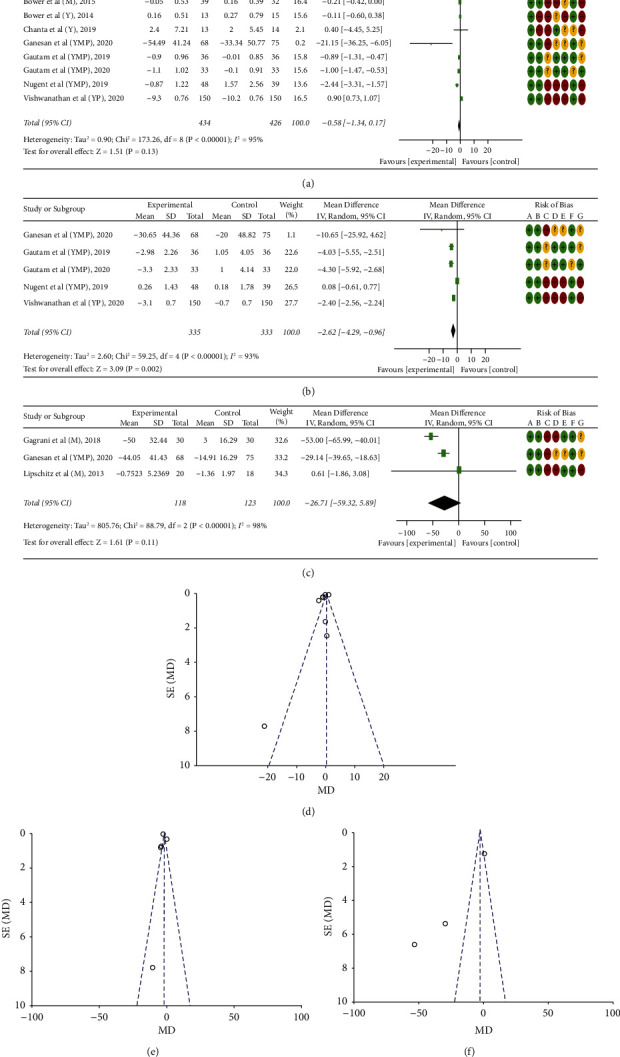
Forest plots including risk of bias (ROB) (a, b, and c) and funnel plots (d, e, and f) of effects of yoga (Y), meditation (M) and/or pranayama (P) on various biomarkers among patients; for ROB; A = Random sequence generation (selection bias); B = Allocation concealment (selection bias); C = Blinding of participants and personnel (performance bias); D = Blinding of outcome assessment (detection bias); E = Incomplete outcome data (attrition bias); F = Selective reporting (reporting bias); and G = Other bias.

For meta-analysis, eleven studies [14, 18, 19, 23, 25, 29, 39, 40, 42, 44, 52] only focused on three biomarkers among the patients met the inclusion criteria, so we calculated the mean differences. The mean differences of IL-6, TNF-*α*, and cortisol were −0.58 pg/mL (95% CI (−1.37, 0.17)) [14, 23, 25, 29, 39, 40, 42, 44, 52], −2.62 pg/mL (95% CI (−4.29, −0.96)) [23, 25, 39, 40, 42], and −26.71 ng/ml (95% CI (−59.41, 5.99)) [18, 19, 25], respectively. Considerable heterogeneity was observed between the studies (IL-6 (*I*^*2*^ = 95%, *p* < 0.000001), TNF-*α* (*I*^*2*^ = 93%, *p* < 0.000001), and cortisol (I^2^ = 98%, *p* < 0.00001) (Figure 2).

#### 3.6.9. Subgroup Analysis

In subgroup analysis (Table 4), yoga-pranayama-meditation (*p*=0.002), 6–12-week (1000–2000 min) intervention (*p*=0.0006), and 8–12-week (3000–4800 min) were found with an overall effect significant in IL-6, cortisol, and TNF-*α*, respectively. In the meditation subgroup of IL-6, a marginal overall effect (*p*=0.05) with no heterogeneity (*I*^*2*^ = 0%) was observed.

#### 3.6.10. Sensitivity Analysis

In a sensitivity analysis, we found a significant overall effect (*p*=0.04) by removing three studies of high risk of bias (C1.2) in IL-6. Similarly, in TNF- *α*, by removing one moderate risk study (B1.3) (*p*=0.003) and two high risk studies (C1.2) (*p*<.00001), overall effects remained significant (Table 5).

#### 3.6.11. Publication Bias

Publication bias was assessed using funnel plots. The majority of the studies were found outside of the 95% CI as visualised, signifying a high publication bias [14, 23, 25, 29, 39, 40, 42, 44] (Figure 2).

## 4. Discussion

**Figure 3:**
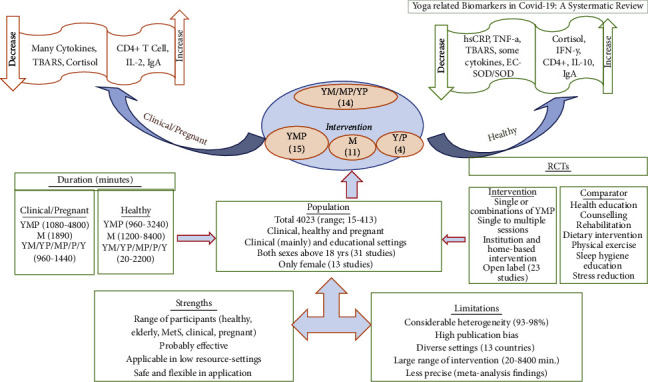
Summary of findings.

The review included 44 studies from 13 countries (including the USA, followed by India, which conducted the maximum RCTs), comprising 4023 people. The studies were conducted in different populations (healthy, diseased, and pregnant) ranging from 20 to 4800 minutes. Our main findings demonstrate that yoga, meditation, and pranayama, either alone or in combination, are effective in improving immunity in healthy and clinical populations (including pregnant women) by regulating anti- and pro-inflammatory biomarkers. Key findings (which were statistically significant) are (i) among clinical participants, there was a decrease in IL-6 [18, 23, 32, 40, 42, 53], IL-1*β* [31], IL 17/17A [40, 42], IL- 1*α* [25], CRP [39, 41], hsCRP [32], TNF/TNF- *α* [23, 25, 35, 40, 42, 46, 53, 55], sTNFR II [29], cortisol [18, 25, 31, 53, 56], TBARS [23]; and an increase in CD4+ T cells (nonsignificant) [33, 36], IL-2 [44], FEV1, PEF, VT, and FVC [55]; (ii) among healthy participants, an increase in IL-10 [37], IFN-*γ*, cortisol [47], and IgA [51]; and a decrease in IL-6 [24, 45], CRP [15], TNF-*α* [24, 26], hsCRP [24], EC-SOD/SOD [32], TBARS [45, 47], MCP-1 [49], and IL-1*β* [26, 37] was observed (iii) among the pregnant women, the intervention was effective in increasing IgA and decreasing cortisol levels [56] (Figure 3).

Many studies have reported that fluctuations in biomarkers among COVID-19 patients play vital roles in immunity [58–67] (Table 6).

In a case report, a 55-year-old COVID-19 positive with comorbidities had his COVID-19 symptoms alleviated as well as his blood sugar level improved after treatment with ayurvedic medicine, yoga, dietary suggestions, lifestyle modifications, and allopathic medications [68]. Another ongoing study can strengthen the importance of yoga intervention in improving the health of COVID-19 patients [69].

Many studies have explored various immunity biomarkers in COVID-19 patients. T cells, an important component of lymphocytes responsible for robust immunity, are reduced significantly during severe infections, including COVID-19 [58, 59]. Cytokines (IL-6, IL-10, IL-8, IL-2R, and so on) consist of various biomarkers produced by both innate and adaptive immune cells [70], which play a vital role in inflammation [71]. A fatal cytokine storm is usually observed in COVID-19 patients who are critically ill [72]. Cytokine storms lead to damaged tissues, resulting in thrombotic tendency and multiple-organ failure [73], suggesting cytokine control necessary. Both individual studies and systematic reviews indicate the importance of IL-6, reporting it to be an independent predictor of disease severity and survival in COVID-19 patients [74–77]. CRP, a prominent marker of systemic inflammation, was elevated in the majority of COVID-19 patients with severe illness compared with mild or nonsevere patients [78–80]. Cortisol regulation acts as an adaptive immunity, but extreme cortisol responses (relative adrenal insufficiency) are associated with a higher mortality rate [81–83]. Cortisol levels were high in COVID-19 positive patients compared to negative [84] and nonfatal [85] COVID-19 patients.

Although meta-analyses showed an overall decrease in stress biomarkers (IL-6, TNF-*α*, and cortisol), only TNF-*α* was significant. However, subgroup analysis showed TNF-*α* (8–12 wk, 3000–4800 minutes), IL-6 (yoga-meditation-pranayama), and cortisol (6–12 wk, 1000–2000 minutes) statistically significant reductions favouring intervention. Our findings are in line with earlier conducted meta-analyses in which both IL-6 and TNF-*α* were reduced in the intervention group following the yoga and meditation [86, 87]. The change was significant in one study reporting IL-6 [86], whereas there was an insignificant decrease in IL-6 and TNF-*α* [87], which could have been due to smaller number of studies (*n* = 2) included in the analysis. Recent reviews on the effects of phytosterols and the effect of an herb, *Nigella sativa*, have also been shown to reduce the cytokine storm; however, these are beyond the scope of this review and so, further suggested [8, 88].

In the scarcity of RCTs reporting the effectiveness and efficacy of yoga, meditation, and pranayama in COVID-19 patients, our findings further add to the yoga-based interventions and their effects on the inflammatory biomarkers. Through our findings, it may be possible to recommend treatment strategies to promote the health of both mild to moderate and severe cases, including symptomatic and asymptomatic COVID-19 patients.

The Indian Public Health Standards (IPHS), in its revised set of standards released in 2012, has integrated yoga as one of the components to be prescribed for AYUSH facilities [89]. Our findings strengthen the usefulness of yoga and its fraternity, which is in tandem with the recommendation of IPHS to implement yoga in primary and secondary health facilities. A minimum of an hour of intervention following a higher duration of yoga followed by pranayama and meditation in healthy and asymptomatic COVID-19 patients can have a positive impact on their health. As COVID-19 patients become more severe, pranayama should be practised for longer periods, followed by meditation and yoga, as various asanas in yoga might be complicated to perform in such patients. Our findings suggest that the intervention can have a long-term positive effect even if practised at home, but beyond 8 weeks, participants might feel unmotivated to continue. Therefore, post-8-week refresher training might be granted as food for thought.

Despite this, the review has certain limitations. Because the studies and participants were from various settings and countries, significant heterogeneity was observed in the meta-analyses, which may cast doubt on the summary effects to some extent and should be approached with caution. Moreover, due to the low number of studies included in meta-analyses, it was unwise and so restricted to carry out quantitative analysis such as Egger's or Begg's tests for publication bias. In the systematic review, we included seven pilot and feasibility RCTs, which may have compromised the internal validity of the review. Furthermore, only a single study was respiratory-related (acute respiratory distress syndrome, ARDS) as a disease outcome; it should be extrapolated in COVID-19 patients with solicitude.

## 5. Conclusion

The systematic review of 44 studies revealed 25 immunity biomarkers, among healthy, clinical, and pregnant women, with possible implications for yoga-related interventions in COVID-19. Total doses of yoga, meditation, or pranayama ranged between 20 and 8400 minutes. However, a smaller dose of YMP was found to make significant changes among pregnant or clinical patients in comparison to healthier ones. Clearly, the combined action of YMP may be effective in lowering IL-6, cortisol, and TNF-*α* levels in patients, specifically with 2625, 3900, and 1500-minute interventions, respectively, spanned over 8–12 weeks. Also, the YMP package is warranted for pilot RCT among COVID-19 mild to moderate patients with standard care before reaching a firm conclusion. Furthermore, meta-analyses of biomarkers and phytosterols of COVID-19 patients are highly recommended.

## Figures and Tables

**Table 1:** Characteristics of included studies.

SN	Author, (1st) year, and country	Intervention type	Title of the article	Study type	Participants (per-protocol)		Experimental intervention	Control intervention	Population characteristics (disease/healthy/ and clinical)	Population characteristics (age and sex)	Biomarkers
					Experimental group	Control group					
1	Zgierska et al., 2016, USA [49]	M	Mindfulness meditation and cognitive behavioral therapy intervention reduces pain severity and sensitivity in opioid-treated chronic low back pain: Pilot findings from a randomized controlled trial	26-week parallel-arm pilot randomized controlled trial (open-label)	21 (21)	14 (14)	Meditation and cognitive-behavior therapy and usual care	Usual care alone	Chronic low back pain (CLBP) with reported having daily CLBP (lum-bosacral area pain or “sciatica” leg pain) and treated with minimum of 30 mg/day of morphine-equivalent dose (MED)	Minimum of 21 years old, on average 51.8 ± 9.7 years old, 80% white women.	CRP, IL-1*ß*, TNF-*α*, IL-6, and IFN-*ץ*

2	Bower et al., 2015, USA [14]	M	Mindfulness meditation for younger breast cancer survivors: a randomized controlled trial	6-week single centre two-arm RCT (open-label)	37(30)	28 [22]	Mindful awareness practices	Wait-list control	Diagnosed with stage 0 – III breast cancer at or before age 50 and completed local and/or adjuvant cancer therapy (except hormonal therapy) at least 3 months prior	Age mean (range) Maps: 46.1 (28.4–60); Control: 47.7 (31.1–59.6)	CRP, IL-6, sTNFRII

3	Meyer et al., 2019, USA [15]	M	Differential reduction of IP-10 and C-reactive protein via aerobic exercise or mindfulness-based stress-reduction training in a large randomized controlled trial	8-week three armed (two intervention and a control), matched controlled arm (single-blinded)	Aerobic exercise, 137 (126); MBSR medication, 138 (127)	138 (132)	Two arms; aerobic exercise and MBSR	Wait-list observational control	Generally healthy and reported to be inactive	Age, all, 50 ± 12, meditation; 49 ± 11, exercise 49 ± 11; control 51 ± 12; male all, 92, meditation 32, exercise 27, control, 33	IL-6, CRP and interferon-gamma-inducible protein-10 (IP-10)

4	Dada et al., 2018, India [53]	MP	Mindfulness meditation reduces intraocular pressure, lowers stress biomarkers, and modulates gene expression in glaucoma: a randomized controlled trial.	21 days prospective, single blinded, randomized controlled trial (single-blinded)	45 (40)	45 (42)	Mindfulness-based stress reduction and meditation group	Wait-list control	Outpatient with primary open angle glaucoma (POAG)	Intervention: 20 females, mean age 57.88 ± 8.17 years control: 20 females, age 56.63 ± 7.12	Cortisol, IL-6, TNF-a at baseline and post intervention

5	Hayney et al., 2014, USA [16]	M	Age and psychological influences on immune responses to trivalent inactivated influenza vaccine in the meditation or exercise for preventing acute respiratory infection (MEPARI) trial	8 weeks 3-experimental arm randomized control trial (single-blinded)	Meditation 51 (51), exercise 47 (47)	51 (51)	(i) MBSR (ii) Exercise	Control group	Healthy individuals were recruited	MBSR : male 9, mean age 60.0 (6.5) years; control: male 10, mean age 58.8 (6.8) years; exercise: male 8, mean age 59.0 (6.6) years	IGA, IL 10, interferon*γ* at baseline and 3 week

6	Barrett et al.,2012, USA [17]	M	Meditation or exercise for preventing acute respiratory infection: a randomized controlled trial	8-week randomized 3 parallel group trial (open-label)	Meditation: 51 (51) Exercise: 51 (47)	52 (51)	(i) Mindfullness meditation (ii) Moderate-intensity exercise	Observational control	Community-based 50 years or older	82% female, 94% white, mean age 59.3 ± 6.6 years	Interleukin-8 levels within 3 days of symptoms

7	Gagrani et al., 2018, India [18]	M	Meditation enhances brain oxygenation, upregulates BDNF, and improves quality of life in patients with primary open angle glaucoma: a randomized controlled trial	6 weeks prospective randomized control trial (open-label)	30 (30)	30 (30)	Meditation with standard medical treatment	Standard medical treatment	POAG (primary open angle glaucoma) patients	21 females and 39 males, mean age of 57.28 ± 9.37 years	Serum cortisol and serum IL-2 & IL-6 at baseline and post intervention

8	Lipschitz et al., 2013, USA [19]	M	Reduction in salivary *α*-amylase levels following a mind-body intervention in cancer survivors—an exploratory study	8-week 3 experimental arm RCT (open-label)	Mbb, 19; Mm, 20	18	One of two experimental mind-body interventions, namely, mind-body bridging (MBB) or mindfulness meditation (MM)	Sleep hygiene education (SHE)	Cancer survivors with any sleep disorder/problem visiting health facility	All, age 18–75, M14, F43; SHE, 52 ± 11, M4, F14; MBB, 55 ± 10, M6, F13; MM, 51 ± 9, M4, F16	Salivary cortisol at baseline and 4th week with two parameters (waking, and daily output

9	Rao et al., 2017, India [20]	M	Effect of yoga on sleep quality and neuroendocrine immune response in metastatic breast cancer patients	3-month RCT (single-blinded)	45 (25)	46 (21)	Integrated yoga-based stress reduction program	Education and supportive therapy sessions	Stage IV breast cancer within 6 months–2 years after diagnosis	Yoga group: average age 48.9 (9.1) years control: 50.2 (9.2)	Cortisol for 3 consecutive days (at 0600 h, 0900 h, and 2100 h)

10	Creswell et al., 2016, USA [50]	M	Alterations in resting state functional connectivity link mindfulness meditation with reduced interleukin-6: a Randomized controlled trial	4 weeks randomized control trial (single-blinded)	18 (17)	17 (17)	Health enhancement through mindfulness (HEM)	Health enhancement through relaxation (HER)	Right-handed unemployed job-seeking community adults	Hem : mean age 37.94 (10.96), 7 female and 11 male Her : mean age 41.00 (9.55), 8 female and 9 male	IL-6

11	Andres-Rodriguez et al., 2019, Spain [52]	M	Immune-inflammatory pathways and clinical changes in fibromyalgia patients treated with mindfulness-based stress reduction (MBSR) : a randomized, controlled clinical trial	8 weeks randomized, controlled clinical trial (single-blinded) with 12 months follow-up	15 (14)	16 (14)	MBSR + TAU	Treatment as usual (TAU)	Female patients with fibromyalgia (FM) diagnosis according to ACR 1990	Age, control, 52 ± 8, intervention, 55 ± 8; all female	IL-6, hsCRP, IL-10 and hsCRP, and their different ratios

12	Buijze et al., 2019, Netherlands [21]	MP	An add-on training program involving breathing exercises, cold exposure, and meditation attenuates inflammation and disease activity in axial spondyloarthritis – a proof of concept trial	8-week open-label, randomized, one-way crossover clinical proof-of-concept trial (open-label)	Early intervention; 13 (13), late intervention, 11 (11)	11 (11)	Add-on training of breathing, cold exposure, and meditation	No intervention at initial intervention	Patients with a clinical diagnosis of axial spondyloarthritis (axSpA) and fulfilled the ASDAS classification criteria and had active diseases defined as ASDAS>2.1 and a high-sensitive CRP (hsCRP) ≥5 mg/L	M = 15, F = 8; age = 35 ± 7	HsCRP at 0, 4, 8, 24, 28 and 32 weeks

13	Mandal et al., 2021, India [22]	YP	Effect of structured yoga program on stress and professional quality of life among nursing staff in a tertiary care hospital of Delhi—a small scale Phase-II trial	12-week open-label, phase-II RCT (open-label)	58 (19)	52 (32)	Yoga intervention	Wait-list control	Healthy staff nurses working in a tertiary care hospital	Intervention, mean age, 35 ± 8, *M*, 8, F, 50; control, mean age, 33 ± 7, *M*, 22, F, 30, F,	Serum cortisol and hsCRP were assessed at baseline and 12 weeks

14	Viswanathan et al., 2020, India [23]	YP	Effect of yoga intervention on biochemical, oxidative stress markers, inflammatory markers, and sleep quality among subjects with type 2 diabetes in south India: results from the SATYAM project	3-month nonregistered randomized experimental study (open-label)	150 (150)	150 (150)	Yoga intervention	Simple physical exercises	Type 2 diabetes	(i) Control: M-103, F-47, age 52.8 ± 7.0 (ii) intervention: M-93, F-57, age 50.8 ± 8.3	TNF-*α*, IL-6, TBARS, EC-SOD and hsCRP were assessed at baseline and 3 months

15	Shete et al., 2017, India [24]	YP	Effects of yoga training on inflammatory cytokines and C‐reactive protein in employees of small‐scale industries	3-month RCT (open-label)	24 (19)	24 (18)	Yoga	Wait-list control	Healthy male adults	Average age 41.5 (5.2) years	IL-6, TNF-*α*, hsCRP at baseline and post intervention

16	Ganesan et al., 2020, India [25]	YMP	Effects of yoga therapy on disease activity, inflammatory markers, and heart rate variability in patients with rheumatoid arthritis	12-week randomized control trial (open-label)	83 (68)	83 (75)	Yoga therapy with standard medical treatment	Standard medical treatment	RA (rheumatoid arthritis)	YG (yoga group): 63 female participants (92.64%), mean age of 41.33 years CG (control group): 68 female participants (90.66%), mean age of 42.59 years	IL-1 *α*, IL-6, TNF-a, cortisol at baseline and after 12 week

17	Chen et al., 2016, China [26]	YMP	Effects of 8-week hatha yoga training on metabolic and inflammatory markers in healthy and female Chinese subjects: a randomized clinical trial	8-week randomized controlled trial (open-label)	15 (15)	15 (15)	Hatha yoga intervention	No intervention	Healthy female participants	Age, 18–25 years, all females	MCP-1, TNF-*α*, IL-6, IL-8, and IL-1*β*

18	Kiecolt-glaser et al., 2014, USA [27]	YP	Yoga's impact on inflammation, mood, and fatigue in breast cancer survivors: a randomized controlled trial	12-week randomized control trial (single-blinded)	100 (96)	100 (90)	Yoga	Waitlist control	Stage 0 to IIIa breast cancer survivors	Yoga: mean age 51.8 (9.8) years Control: mean age 51.3 (8.7) years	IL-6, TNF- *α*, and IL-1beta

19	Kaminsky et al., 2017, USA [28]	P	Effects of yoga breathing (pranayama) on exercise tolerance in patients with chronic obstructive pulmonary disease: a randomized, controlled trial	12-week randomized control trial (double-blinded)	21 (21)	22([22)	Pranayam plus education	Education	Physician diagnosed COPD (chronic obstructive pulmonary disease) patients with 18 years age or older	Pranayam: female % (67%), mean age 68 (7) Control: female% (55%), mean age 68 (9)	IL-6, CRP, 6MWD, FEV1, IC, rv/tlc, DLCO, va/tlc, Ti/Ttot

20	Bower et al., 2014, USA [29]	Y	Yoga reduces inflammatory signaling in fatigued breast cancer survivors: a randomized controlled trial	12-week randomized intervention (open-label)	16 (14), 13 (3 months follow-up))	15 (15)	Iyengar yoga	Health education control	Breast cancer survivors of stage 0 - II with mean diagnosis of 3.6 ± 3.7 years ago	Average age of 54 ± 5.4 years	sTNF Receptor-II, IL-1 RA, IL-6 and CRP, salivary cortisol

21	Twal et al., 2016, USA [3]	P	Yogic breathing when compared to attention control reduces the levels of proinflammatory biomarkers in saliva: a Pilot randomized controlled trial	20-min pilot randomized controlled trial (open-label)	10 (10)	10 (10)	Yogic breathing (YB)	Attention control (AC)	Normal apparently healthy	5 males and 5 females in both groups. Age ranged from 27 to 33 years	IL-1RA, IL-6, IL-10, IL-17, IP-10, MIP-1b, TNF-*α*, (IL)-1*β*, IL-8, MCP-1 assessed on 0, 5, 10, 15 and 20 minutes after intervention

22	Hopkins et al., 2016, USA [30]	YP	Heated hatha yoga to target cortisol reactivity to stress and affective eating in women at risk for obesity-related illnesses: a randomized controlled trial	8 weeks randomized control trial (single-blinded)	27 (27)	25 (24)	Bikram yoga	Wait-list control	Community females age 25 to 45 years of age	Females with mean age 33.5 (6.4) years	Cortisol at baseline and post intervention

23	Banasik et al., 2011, USA [31]	YP	Effect of iyengar yoga practice on fatigue and diurnal salivary cortisol concentration in breast cancer survivors	8-week randomized controlled trail (open-label)	9 (7)	9 (7)	Iyengar yoga	Wait-list control, no intervention	Stage II–IV breast cancer survivors	All womens were caucasian; control group age: 62.4 ± 7.3 yoga group age:63.33 ± 6.9	Salivary cortisol at baseline and 8 weeks

24	Marques et al., 2017, Portugal [54]	YP	Influence of chair-based yoga on salivary antimicrobial proteins, functional fitness, perceived stress and well-being in older women: a Randomized pilot controlled trial	28 weeks RCT (open-label)	15 (15)	10 (10)	Chair based yoga (CBY)	Control group	Older women living in two different health and social caregiver centres (HSC)	EG = average age 83.73 (6.86) years GC = average age 82.73 (8.46) years	IgA and cortisol were assessed at baseline and 28 week

25	Torkamani et al., 2018, Iran [51]	M	Effects of single-session group mantra-meditation on salivary immunoglobulin and affective state: a psychoneuroimmunolgy viewpoint	20 minutes RCT (single-blinded)	15	15	Meditation	Control	Healthy women	Mean age 44 ± 3 years	s-IgA at baseline, after lecture, post-meditation and one hour later

26	Gunjiganviet al., 2021, India [55]	YP	Yoga—an alternative form of therapy in patients with blunt chest trauma: a randomized controlled trial	4-week RCT (open-label)	46 (32)	43 (27)	Yogatherapy (YTP) with standard chest physiotherapy (CTP)	Chest physiotherapy (CTP)	Patients aged 18–65 years with isolated blunt chest trauma and who were managed nonoperatively with or without thoracostomy tubes	Intervention: male 40, mean age 41.1 (12.40) years; control: male 36, mean age 42.6 (11.59)	Serum levels of ILs TNF-a, IFN-c; along with respiratory indicators assessed

27	Pullen et al., 2010, USA [32]	YM	Benefits of yoga for african american heart failure patients	8-week RCT (single-blinded)	21 (18)	19 (16)	Hospital-based yoga group	Control group (CG, standard medical care)	Systolic or diastolic heart failure (HF) patients of ischemic or nonischemic etiology	Yoga: mean age 55.8 (±7.6); control: mean age 52.5 (±12.7) years	IL-6, hsCRP, and EC-SOD baseline to 8 weeks

28	Hecht et al., 2018, USA [33]	YM	A randomized, controlled trial of mindfulness-based stress reduction in HIV infection	8-week single center, randomized controlled parallel trial (single-blinded)	89 (76 at 3 month & 48 at 12 month)	88 (76 at 3 month & 48 at 12 month)	MBSR	Education control	18 years of age or older with HIV-1 infection	MBSR : median age of 41 (22–63) years; control: median age of 39 (22–66) years	CD4+, hsCRP, IL-6 at baseline, 3 month and 12 month

29	Nijjar et al., 2019, USA [34]	YMP	Randomized trial of mindfulness-based stress reduction in cardiac patients eligible for cardiac rehabilitation	8-week pilot randomized controlled trial (open-label)	31 (30)	16 (15)	Mindfulness-based stress reduction (MBSR)	Usual care control	CR-eligible (exercise-based cardiac rehabilitation) cardiac patients	Age, all, 61 ± 11, MBSR, 59 ± 12, control, 61 ± 9;	hsCRP at baseline, 3 month, and 9 month

30	Huberty et al., 2019, USA [35]	YM	Online yoga in myeloproliferative neoplasm patients: results of a randomized pilot trial to inform future research	12-week pilot RCT (single-blinded)	34 (27)	28 (21)	Online yoga	Wait-list control	Myeloproliferative neoplasm (MPN) patients b) reported engaging in ≥150 min/week of physical activity	Yoga: female 25, mean age 58.3 (9.3) years; control: female 20, mean age 55.0 (11.4)	IL-6 and TNF-*α* were assessed at baseline and 12 week

31	Chen et al., 2017, taiwan [56]	YMP	Effects of prenatal yoga on women's stress and immune function across pregnancy: a randomized controlled trial	20-week prospective RCT (open-label)	50 (48)	51 (46)	Routine prenatal care plus yoga intervention	Routine prenatal care	Preganant women from 16 to 36 week's of GA	Mean chronological age 33.0 ± 3.8 years (range = 24–43)	Salivary cortisol and IgA, and CD4+T cell

32	SeyedAlinaghi et al., 2012, Iran [36]	YM	RCT of mindfulness-based stress reduction delivered to HIV + patients in Iran: effects on CD4+ T lymphocyte count and medical and psychological symptoms	8-week randomized control trial (single-blinded)	120 (87)	125 (86)	Mindfulness-based stress reduction (MBSR)	Education and support (ESC)	18+ years and HIV + confirmed by western blot.	Mean age was 35.1(SD = 6.5) years and 69% were male	

33	Rajbhoj et al., 2015, India [37]	YMP	Effects of yoga module on proinflammatory and anti-inflammatory cytokines in industrial workers of lonavla: a randomized controlled trial	12-week RCT (open-label)	23 (19)	22 (18)	Yoga	Wait-list control	Industrial workers	Yoga group: mean age 40.72 ± 6.79 age; control group: mean age 40.18 ± 6.31 age	IL-1*β*, IL-10 at baseline and post intervention

34	Singh et al., 2011, India [38]	YMP	Effects of yogic package on rheumatoid arthritis	7 weeks days RCT (open-label)	40	40	Yoga and usual meditation	Usual medical care	Rheumatoid arthritis	Age, intervention, mean 35.1 (±7.3), control, mean 34.7 (±7.3) yrs	CRP at baseline and post intervention

35	Nugent et al., 2019, USA [39]	YMP	Benefits of yoga on IL-6: findings from a randomized controlled trial of yoga for depression	10-week RCT (single-blinded)	48 (48)	39 (39)	Hatha yoga intervention (yoga)	Healthy living workshop (HLW)	Patients of major depressive disorder with age 18 years or older	Age, yoga, 46 ± 13, HLW, 45 ± 14, total, 45 ± 13; sex, yoga, M4, F44, HLW, M10, F29,	IL-6, CRP and TNF-*α*

36	Gautam et al., 2019, India [40]	YMP	Impact of yoga-based mind-body intervention on systemic inflammatory markers and co-morbid depression in active rheumatoid arthritis patients: A randomized controlled trial	8-week randomized control trial (single-blinded)	36 (30)	36 (32)	Yoga-based mind-body-based intervention with usual medical care	Usual medical care	Outpatient of RA (rheumatoid arthritis)	Age, yoga, mean 45.7(±1.6), control,,control, mean 42.1(±1.7) yrs	IL-17 A, IL-6, TNF-*α* at baseline and 8 week

37	Sohl et al., 2016, USA [41]	YMP	A brief yoga intervention implemented during chemotherapy: a randomized controlled pilot study	8 weeks RCT (open-label)	8 (6)	7 (5)	Yoga skill training (YST)	Attention control (AC)	Colorectal cancer stage 0-IV	Median age 61.0 (44.0 to 67.0) years, male 9	IL-6, IL-1RA, sTNF Ri, TNF-*α*, CRP

38	Gautam et al., 2020, India [42]	YMP	Effects of an 8-week yoga-based lifestyle intervention on psycho-neuro-immune axis, disease activity, and perceived quality of life in rheumatoid arthritis patients: a randomized controlled trial	8-week RCT (single-blinded)	33 (31)	33 (31)	Yoga-based lifestyle intervention (yoga group)	Usual care control (non-yoga group)	Patients of rheumatoid arthritis	Age, yoga, mean, 45.1 (±8.7), F28 control, mean 43.4 (±9.3) yrs, F25	IL-6, IL-17A, TNF-*α* at baseline and 8 week

39	Wolff et al., 2015, Sweden [43]	YMP	Yoga's effect on inflammatory biomarkers and metabolic risk factors in a high risk population – a controlled trial in primary care	12-week three armed (two intervention and a control), matched controlled open clinical trial (open-label)	Group 1, 28 (21); group 2, 28 (20)	27 (22)	Yoga intervention (group 1) and yoga instruction (group 2) for home practice	Standard care	Adults diagnosed with hypertension	Age Ex1, 66, F19, Ex2, 64, F20, control, 61, F16, respectively	HsCRP and IL-6 were measured at baseline and 12 weeks

40	Chanta et al., 2019, Thailand [44]	Y	Effects of hatha yoga training on rhinitis symptoms and cytokines in allergic rhinitis patients	8-week randomized controlled trial (single-blinded)	15 (14)	15 (13)	Hatha yoga (YOG)	Normal activities (CON)	Allergic rhinitis patients visiting university health service center	Age, 18–45 yrs; sex, CON, 14 (3 male, 11 female); YOG, 13 (3 male, 10 female)	IL-2 and IL-6 were assessed from nasal secretions at baseline and 8 week

41	Yadav et al., 2018, India [45]	YMP	Comparative efficacy of a 12-week yoga-based lifestyle intervention and dietary intervention on adipokines, inflammation, and oxidative stress in adults with metabolic syndrome: a randomized controlled trial	12-week parallel, two arm, RCT (open-label)	130 (89)	130 (79)	Two interventions were carried out. Firstly, yoga-based lifestyle intervention (YBLI) and secondly, dietary intervention (DI)	Only dietary intervention (DI)	Younger apparently healthy adults, diagnosed with metabolic syndrome	Aged 20–45 years, both males and females	TNF-*α*, IL-6, TBARS, SOD assessed at baseline, 2 weeks and 12 weeks

42	Harkess et al., 2016, austrailia [46]	YMP	Preliminary indications of the effect of a brief yoga intervention on markers of inflammation and DNA methylation in chronically stressed women	8 weeks randomized control trial (single-blinded)	11 (11)	15 (15)	Yoga group	Control group	Women reporting psychological distress	Mean age 41.12 (4.28) years	IL6-1, IL6-2, hsCRP, TNF

43	Lim at al., 2015, Republic of Korea [47]	YMP	Regular yoga practice improves antioxidant status, immune function, and stress hormone releases in young healthy people: a randomized, double-blind, controlled pilot study	12-week randomized double blind control trial (double-blinded)	12 (12)	13 (13)	Yoga	Control group	Young healthy university student	Control: median age 22 years, 8 women yoga: median age 21 years, 6 women	Serum lipid peroxide level, TNF-a, IFN--*γ* and IL-12, EC-SODs, and cortisol

44	Jorge et al., 2016, Brazil [48]	YMP	Hatha yoga practice decreases menopause symptoms and improves quality of life: a randomized controlled trial	12-week 3 experimental arm randomized control trial (single-blinded)	Yoga 47 (40), exercise 38 (29)	32 (19)	(i) Yoga (ii) Exercise	Control	Healthy yoga-naive women between 45 and 65 years	Yoga: mean age 54 (6) years; exercise: mean age 56 (5) years; control: mean age 55 (4)	Salivary cortisol at baseline and at 12 week intervention

**Table 2:** Details of included studies.

SN	Author (1st), year, country	Intervention type	Intervention details	Key findings (descriptive)	Strengths and limitations
1	Zgierska et al., 2016, USA [49]	M	(i) Included different meditations including mini-meditation, loving kindness meditation, and breathing meditations relating to pain supported by psychologists, (ii) In addition participants were encouraged to practice formal mindfulness meditation through the duration of the study	(i) Changes in Cohen's d of CRP, IL-1*ß*, TNF-*α*, IL-6, and IFN-ץ ranged from 0.05 to 0.53 and -0.16 to −0.51 (minimal to medium) (ii) Nonetheless all of these changes were statistically insignificant (*p* > 0.05)	(i) Statistically insignificant but from small to moderate effects, indicate further study (ii) The combined package of meditations with breathing exercises may be explorative

2	Bower et al., 2015, USA [14]	M	(i) Mindful awareness practices (MAPs) for 6 weeks including 2 hours group session (ii) Consisting of experiential practice of meditation and gentle movement exercises (e.g., mindful walking) (iii) Psychoeducation (iv) Home practice of mindfulness technique on daily basis beginning with 5 minutes per day increasing to 20 minutes per day	(i) CRP level, IL-6, and sTNFRII, all decreased in intervention groups but were statistically nonsignificant	(i) wait-list control has not controlled for nonspecific effects of the intervention

3	Meyer et al., 2019, USA [15]	M	(i) MBSR (intervention group) consisted of 2.5-hr sessions once per week for 8 weeks with an additional “half-day retreat.“, and practice either formal or informal meditation at home each day, (ii) exercise (control group) consisted a 2.5-hour session once per week for 8 weeks	(i) CRP changed with small (ii) IP-10 changed minimally	(i) Randomizing participants (exercise and meditation) may capture the comparative immunity benefits

4	Dada et al., 2018, India [53]	MP	(i) Daily group meeting for 60 minutes. (ii) Participants introduced to slow and deep breathing exercise from day 2 (till 21 days). (iii) Every day, 15 minutes relaxation, and 45 minutes meditation was executed	(i) The cortisol level, IL-6, and TNF-*α* decreased postintervention significantly.	(i) There was no significant difference in the biomarker in intervention and control group and baseline.

5	Hayney et al., 2014, USA [16]	M	(i) The MBSR included weekly 2.5-hour group session and 45 minutes of daily at home practice (ii) The exercise included weekly 2.5-hour group session and 45 minutes of daily at home practice	(i) IgA decreased in the meditation group (ii) Median interferon *γ* also increased in meditation group (iii) Median Interleukin-10 production showed a overall increase in meditation group	(i) Statistical analysis between group and within group change in biomarkers at baseline and endline is not carried out

6	Barrett et al.,2012, USA [17]	M	(i) Meditation group, was given, weekly twice 1⁄2-hour group sessions and 45 minutes of daily at home practice, focusing on stress manifestations which may lead to a healthier mind-body response. (ii) Exercise group was given, weekly twice a 1⁄2-hour group session of didactic instruction and moderately intensive exercise using stationary bicycles, treadmills, and other equipment, and 45 minutes of daily at home practice of brisk walking or jogging	(i) IL-8 means of meditation and exercise groups were 252 pg/ml and 36 pg/ml higher than that of control group (postintervention).	(i) Two cohort were selected at different time point which might influence the results. (ii) Sample size was marginal for statistical significance

7	Gagrani et al., 2018, India [18]	M	(i) Daily 45 minutes session was held in which participants sit on the floor and close their eyes to focus on breathing	(i) The mean serum cortisol level decreased in the intervention (*p*=0.01) (ii) The mean serum IL-6 level decreased in (*p* < 0.001)	(i) Sample size was probably non-normal and insufficient (convenience sampling)

8	Lipschitz et al., 2013, USA [19]	M	(i) Mindfulness-meditation (MM) included the breath meditation, body-scan meditation, walking meditation, and forgiveness meditation (ii) Sleep health education (SHE) included ways to change daily activities and habits to improve their sleep (iii) Mind-body bridging (MBB) included expanding awareness to face daily life challenges by reducing stress and addressing sleep issues.	(i) Waking cortisol decreased (ns) (ii) MBB slightly outperformed (with producing higher salivary cortisol amount) compared to MM and SHE intervention.	(i) Small sample size with a single day collection and assessment (ii) The difference of changes in the control group and the two intervention groups is very minimal, so providing very weak evidence to conclude

9	Rao et al., 2017, India [20]	M	(i) Integrated yoga-based stress reduction program included 60-min two times/week for 12 weeks and consisted of a set of asanas (postures performed with awareness) breathing exercises, pranayama, meditation, and yogic relaxation techniques. (ii) Participants were also asked to practise at home and maintain a diary (iii) Control intervention was given to supportive counselling sessions, including education and reinforcing social support	(i) Significant decrease in the cortisol level in intervention group was observed only at 0600 hr pre post-intervention (*p* < 0.05) (ii) The mean cortisol level decreased in both the intervention and control groups but insignificant	(i) Active control group (ii) The sample for biomarkers were collected at three time point

10	Creswell et al., 2016, USA [50]	M	(i) Health enhancement through mindfulness (HEM) included mindfulness-based stress reduction consisting of mindfulness training through body scan awareness exercise, sitting and walking meditations, mindful eating, mindful stretching, and discussion (ii) Health enhancement through relaxation (HER) included positive treatment expectancies, group support, teacher attention, physical activity, and mental engagement.	(i) Mean raw IL-6 level decreased in the HEM group from baseline to 4 month follow-up, (ii) mean raw IL-6 level increased in the HER group from baseline to 4 month follow-up	(i) Participants'retainment was good (ii) Home practice was not followed and so, not effective

11	Andres-Rodriguez et al., 2019, Spain [52]	M	(i) Mindfulness-based stress reduction (MBSR) included 8 weekly sessions of around 2.5 h each for mindfulness exercises, with home mindfulness practice (45 min/day) and intensive mindfulness meditation retreat of 6 hours (ii) Treatment as usual (TAU) included the pharmacological and adjusted to the fibromyalgia (FM) patients' symptomatic profile and counselling about aerobic exercise	(i) IL-10 decreased postintervention in TAU and increased in MBSR group (*p*=0.034) with high effect size (ES = −0.72) (ii) IL-6 increased postintervention in both groups (iii) HsCRP, decreased in both groups	(i) Low sample size under powers the study

12	Buijze et al., 2019, Netherlands [21]	MP	(i) 8-week add-on training program consisted of breathing exercises for an average of 30 breaths and strength exercises (e.g., push-ups and yoga balance techniques), gradual cold exposure with immersing whole-body in ice-cold water (0–1°C) for several minutes, and meditation with the eyes closed for 15–20 minutes (ii) During the first 4 weeks, participants had group trainings twice weekly, the second 4 weeks once weekly.	(i) HsCRP progressively decreased and increased in intervention and control group in both endpoints, but remained nonsignificant in both groups (*p* > 0.05)	(i) Not powered to investigate efficacy (ii) The adherence to the group sessions was reported to be high

13	Mandal et al., 2021, India [22]	YP	(i) Consisted of asana, pranayama, and deep relaxation technique (ii) Included 5 minutes deep relaxation technique practiced in supine position, shavasana (corpse pose) to relax the whole-body completely within a short amount of time (iii) Two sessions in a week each with a duration of 50 minutes for 12 consecutive weeks were conducted	(i) Although negative standardized mean difference (SMD) values were obtained for cortisol and hsCRP, both remained nonsignificant (*p*=0.112, 0.784), respectively	(i) Both the per-protocol and intention to treat analysis was conducted. (ii) Association of stress with cortisol was observed demanding further exploration

14	Viswanathan et al., 2020, India [23]	YP	(i) Yoga included practice of asanas such as thadasana, trikonasana, vajrasana, konasana, patchimothasana, uttanapadasana, sarvangasana, matchyasana, salabasana, and pranayama including abdomen breathing, nadisudhi, kabalbhati, sitali, and brahmari relaxation technique, for a period of 50 min for 5 days in a week for 3 months. (ii) Exercise (control) group included simple physical exercises for 50 min for 5 days in a week for 3 months.	(i) TBARS, hsCRP reduced while SOD increased in the control arm, whereas IL-6 and TNF-*α* decreased in the intervention group.	(i) Lack long-term follow- up. May be the first study with a large sample size that nonyoga group in a tertiary care centre for diabetes. (ii) High response rate (75%)

15	Shete et al., 2017, India [24]	YP	(i) 1 h of yoga was carried out, with various (>20) poses, including warm up, asanas, and pranayama, per day, 6 days a week, divided into three stages, namely, Adaptation stage, advancement in yoga practice, and continuation stage (ii) Pranayama included anulom vilom, bhramari, ujjayi, and kapalabhati	(i) Hs-CRP (*p* < .01), IL-6 (*p* < .001), and TNF-a (*p* < .001) significantly reduced in the YG after 12 weeks of yoga compared to control	(i) The finding cannot be generalized due to selection bias and small sample size

16	Ganesan et al., 2020, India [25]	YMP	(i) A total of 30 min; 3 times/week for 12-week yoga therapy was carried out in a research centre with warming up yoga exercises, followed by different (7) yogasanas then followed by pranayama and then, dhyana	(i) IL-6 and TNF- *α* decreased in the control group whereas IL- 1 *α*, cortisol, IL-6 and TNF- *α* decreased in the intervention group, both significantly (ii) In between group comparison all biomarkers decreased but insignificantly.	(i) The biomarkers level where comparable at baseline (*p* > 0.05). (ii) Observation was conducted only at baseline and after 3 months. (iii) Patients with low-to-severe disease activity were included in the study.

17	Chen et al., 2016, China [26]	YMP	(i) Hatha yoga sessions included twice per week over the 8 wks consisting each session of 60 minutes with breathing exercise, loosening exercise, followed by different poses. These poses were followed by relaxation/corpse pose and seated meditation.	(i) TNF-*α* and IL-6 decreased significantly between and within the group (ii) IL-1*β* also decreased in the yoga group (*p* < 0.05)	(i) Study is focused on metabolic syndrome (MetS) among healthy females (ii) Withholds a good retention rate

18	Kiecolt-glaser et al., 2014, USA [27]	YP	(i) Yoga group included two 90-minute sessions per week with different poses in different positions, for 24 sessions. (ii) Each session was followed by different four types of breathing practices	(i) All cytokines and TNF-*α* decreased but remained nonsignificant	(i) The attrition level was low (ii) Between group interpretation was not done

19	Kaminsky et al., 2017, USA [28]	P	(i) Pranayam plus education group practised the dirgha three-part breath pranayam yoga learning educational materials for one-hour per session (ii) Education (only) group learned of education material for the whole 60 minutes	(i) in the intervention group CIP levels decreased, whereas IL-6 increased (both insignificantly) (ii) The secondary biomarkers (FEV1, IC, RV/TLC, DLCO) levels changed (insignificantly) in the intervention group	(i) Instead of professional yoga instructors, research coordinator provided training (ii) Double blind controlled trial

20	Bower JE et al., 2014, USA [29]	M	(i) 12-week iyengar yoga included the yoga focusing on postures, passive inversion (upside-down postures), and passive backbends (supported spinal extensions) for 90 minutes twice a week	(i) sTNF RII level decreased in the intervention group (*p*=0.03 ) for both group and time even after controlling the confounders	(i) Inclusion of active control group (ii) Small sample size (iii) In some results, *p* values have not been reported.

21	Twal et al., 2016, USA [3]	P	(i) Yogic breathing (YB) group was given a combination of 10 min of om chanting (pranava pranayama) followed by 10 min of thirumoolar pranayama (TMP), which includes an inhalation (purakam), breath-holding (kumbakam), and exhalation (Resakam) (ii) Attention control (AC) group performed quiet reading for the same period in independent one-on-one sessions	(i) MCP-1, IL-8, and IL-1*β* all, were found decreased after the intervention of yogic breathing, IL-8, and MCP-1 were also with time. (ii) There was no significant difference in the salivary levels of IL-1RA, IL-6, IL-10, IL-17, IP-10, MIP-1b, and TNF-*α*	(i) First to demonstrate feasibility of the salivary cytokines using multiplex assay. (ii) Small sample size (YB = 10; AC = 10)

22	Hopkins et al., 2016, USA [30]	YP	(i) The yoga consisted of standardized series of 26 hatha yoga postures, two breathing exercises, and two savasanas (i.e., a resting/relaxation posture) in a room heated to 104°F (ii) The yoga sessions were at least two 90 minutes session per week for 8 weeks	(i) Mean cortisol level in both groups decreased (more in yoga group) postintervention.	(i) Two groups were heterogenous (ii) Inferential analysis was not performed

23	Banasik et al., 2011, USA [31]	YP	(i) Iyengar yoga was taught which is more physically demanding and has difficult poses. (ii) Props were used to maintain proper alignment and forms during the yoga. (iii) The yoga session was of 90-min twice weekly	(i) The mean salivary cortisol in morning and 5 pm decreased in the intervention group postyoga participation (*p*=0.018 and *p*=0.028, respectively)	(i) Saliva collection could have been biased. (ii) Out of 18 participants only 14 completed the study

24	Marques et al., 2017, Portugal [54]	YP	(i) Three 50-min session, 2-3 times/week divided into three parts, namely, Joint mobilization and exercises to promote respiratory body awareness (10 min), standing or sitting practice of āsanas and postures (30 min), and cool down and relaxation (10 min)	(i) Insignificant increase in the salivary cortisol level between the groups. (ii) Increase in IgA level in the intervention and decrease in the control group both insignificantly	(i) The participants were polymedicated and so, may differently influence the effects of the intervention

25	Torkamani et al., 2018, Iran [51]	M	(i) The participants of both the groups attended the lecture (45 min) on mantra-meditation. (ii) Only the intervention group meditated for 20 minutes	(i) Between group differences in the IgA level was significant at postintervention (*p*=0.0001) and an hour after intervention (*p*=0.0001).	(i) Small sample size. (ii) Participants were earlier enrolled in yoga classes

26	Gunjiganvi et al., 2021, India [55]	YP	(i) Yogatherapy (YTP) included a maximum of 1 hour of pranayama, and then gradually moved to asanas, as tolerated on daily basis till discharge (3–4 days of admission) and then continue for 4 weeks at home. (ii) Standard chest physiotherapy (CTP) included percussion, vibration, cough stimulation techniques, and breathing exercises and mobilization	(i) Mean differences of most of the biomarkers on day 1, 2, and 3, and at week 4 remained nonsignificant except IL-4 in day 1, IL-10 in day 3, TNF-*α* on day 2, and IFN-ץ on day 2. (ii) The intervention was noneffective at 4 week for VT (*p*=0.056) and FVC/FEV1 (*p*=0.30), but was effective for FVC (*p*=0.008), FEV1 (*p*=0.009), and PEF (*p*=0.016)	(i) One of the few studies explaining yogatherapy as an additional rehabilitation strategy in injured patients. (ii) Home practice for 4 weeks may be biased

27	Pullen et al., 2010, USA [32]	YM	(i) One-hour yoga session consisting of breathing exercises (pranayama), standing and seated yoga postures, followed by relaxation with meditation, was conducted twice per week, in a quiet room, for a total of 16 supervised sessions during an 8- to 10-wk period. (ii) Standard medical care was provided to control group (CG)	(i) The levels of hsCRP, IL-6 and EC-SOD decreased significantly in the intervention group compared to the control group.	(i) Long-term follow-up of the patients' adherence to the yoga and walking would be questionable. (ii) Home-based yoga activity track report was difficult to maintain

28	Hecht et al., 2018, USA [33]	YM	(i) MBSR included eight weekly classes of 2.5 hour duration, focusing body scan meditation, gentle yoga for body awareness and sitting meditation. (ii) At sixth week an 8-hour silent retreat was conducted and assignments was provided for home practice. (iii) Educational/control group carried out 1.5 hours of group session each week for 8 weeks that covered a variety of educational topics about managing HIV infection	(i) There was a increase in CD4 T cells, decrease in IL-6 levels, increase in hsCRP level, but all were nonsignificant.	(i) Assessed long-term effects of meditation. (ii) Difficult to monitoring the interventions at home

29	Nijjar et al., 2019, USA [34]	YMP	(i) MBSR consist of mindfulness meditation, breathing practices, and gentle yoga. (ii) Consists of eight 2.5-hour weekly sessions and one 6.5-hour retreat	(i) HsCRP decreased consecutively at both endpoints but remained nonsignificant (*p*=0.093, adjusted difference)	(ii) A small single center pilot study to assess the feasibility. (ii) To be cautiously generalizable

30	Huberty et al., 2019, USA [35]	YM	(i) Online yoga (OLY) intervention included a 60-min/week home-based, online-streamed yoga (online platform for yoga fitness and meditation practice) for 12 weeks, progressively, mild- to moderate-intensity yoga classes based on hatha and vinyasa-style. (ii) Also included the videos for warm-up and cool down, reminders for breathing with the movements/poses, and a closing mindfulness activity and final relaxation	(i) There was a large decrease in TNF-*α* in OLY participants (−1.3 ± 1.5 pg/ml; ES = −0.87, large effect size). (ii) Change in IL-6 (ES = −0.26) was small	(i) Findings had not a well-defined comparator. (ii) The follow-up of 4 weeks is relatively shorter

31	Chen et al., 2017, taiwan [56]	YMP	(i) Six 70-min yoga sessions per week for 20 weeks, with 10–12 women in each session, which included physical postures/stretching, deep breathing, guided imagery, and deep relaxation	(i) Salivary mean cortisol differences at weeks -16, 20, 24, 28, 32, and 36 were significantly decreased (all *p*′*s* < 0.05). (ii) Salivary mean IgA differences at weeks -16, 20, 24, 28, 32, and 36 postinterventions in yoga groups were significantly increased (all *p*′*s* < 0.05)	(i) First study showing the change in salivary cortisol and IgA in pregnant women. (ii) The study was powered to measure the change. (iii) Good retention (85%)

32	SeyedAlinaghi et al., 2012, Iran [36]	YM	(i) MBSR had 14 sessions, 1 hourly spread over 8 weeks (weekly two sessions). (ii) It consisted of sitting meditation, gentle mindful hatha yoga, a body scan meditation, and in the final session, a 6-hour retreat. (iii) Education and support (ESC, control group) group was given educational information and pamphlets about living healthily with HIV/AIDS.	(i) There was a significant increase in the CD4 counts in MBSR group (*p* < 0.001), 3-months (*p* < 0.001), 6-month (*p* < 0.001), and 9 month (*p* < 0.05), but decreased at 12-month	(i) The CD4+ level at baseline was significantly different in both the groups. (ii) Repeated measures were taken

33	Rajbhoj et al., 2015, India [37]	YMP	(i) Each yoga session was conducted for 45 min, six days a week, for 12 weeks, excluding weekly holidays and consisted of 19 different yoga poses, 5 minutes for each, followed by three different breathing exercises and finally, om chanting	(i) The decrease in mean IL-1*β* levels was significant in the yoga group (*p* < 0.05). (ii) There was a significant increase in the mean IL-10 in the yoga group (*p* < 0.05)	(i) The sample size was small. Different types of poses may be difficult to remember to carry on

34	Singh et al., 2011, India [38]	YMP	(i) One and a half hour per day for 7 weeks (i.e. 40 days approximately) excluding Sunday. (ii) Composed of practices (cleansing practices like gayatri mantra (5 min), kunjal (twice/week), jal nethi (thrice/week), ananas (50 min/week), and healthy yoga diet), pranayamas (20 min), and meditation (15 min)	(i) Decrease in the CRP in the YG after 7 weeks of yoga (*p* < 0.01)	(i) Results were promising, and also, reliable

35	Nugent et al., 2019, USA [39]	YMP	(i) Hatha yoga (intervention group) was carried out at least one group class per week for 10 weeks, and included breathing exercises (pranayama) and seated meditation; warm-ups and half sun salutations; standing postures (asanas); seated postures; an inversion and a twist; and shavasana (relaxation) (80 min). (ii) HLW (healthy living workshop) included at least one HLW class (60 minutes long) per week for 10 weeks, addressing alcohol, nicotine, and caffeine; being a smart patient; brain diseases; cancer prevention; diabetes; nutrition (3 classes); germs, colds, and the flu; physical activity (2 classes); sleep; physical pain, prevalence and causes of depression; and protecting your heart	(i) IL-6 levels reduced significantly in the intervention group compared to control. (ii) TNF-a and CRP did not show evident significant change	(i) Sensitivity analysis of the effect on IL-6 and parameter estimates of growth model have been carried out

36	Gautam et al., 2019, India [40]	YMP	(i) Patanjali raj yoga (classical yoga) was taught to the participants for 120 minutes/per day/5 session/8 weeks. (ii) Including a set of different asanas (physical postures), pranayama (breathing practices) and dhyana (meditation)	(i) Between groups the mean reduction in the CRP level, IL- 17A, IL-6, and TNF-*α* in the yoga group from baseline to 8-week was significant (*p* < 0.05)	(i) Active control group could have explained the change in biomarker is due to the intervention or otherwise

37	Sohl et al., 2016, USA [41]	YMP	(i) Taught a set of yoga skills training (YST) by 3 trainers, consisting awareness meditation, movement, and breathing and relaxation. (ii) For home practise an audio recording of YST was provided and asked to practice a 15-minute session (4times/week)	(i) No significant change was observed in the levels of IL-6, IL- 1 ra, TNF- *α*, and CRP (*p*>0.05)	(i) Underpowered sample

38	Gautam S et al., 2020, India [42]	YMP	(i) Yoga-based lifestyle intervention (YBLI) included Patanjali's ashtanga yoga, asanas (physical postures), pranayama (breathing techniques), dhyana (meditation), and savasana (relaxation techniques). 120 min per session, 5 days for 8 weeks. (ii) Usual care group (control) were continued with usual medical care and normal routine activity for 8 weeks	(i) Significant decline was noted in the levels of pro-inflammatory cytokines (IL-6, TNF- *α*, and IL- 17A) in yoga group after 8 weeks	(i) Lacking active control group. (ii) Small sample size and misbalanced male to female ratio, may limit the power of the effects. (iii) Long duration of the intervention

39	Wolff et al., 2015, Sweden [43]	YMP	(i) Group 1 met once a week for 60 min and practiced kundalini yoga consisting of various yoga (30 min) movements and positions, breathing techniques, and meditation. (ii) Group 2 were given a doctor's appointment (20 min), received instructions for two yoga exercises and did the left nostril breathing, followed by spinal flex	(i) The mean change in IL-6 and hsCRP levels in the intervention group were insignificant.	(i) IL-10 was not detectable in a majority of the patients. (ii) The study was carried out in a primary care settings. (iii) The participants were group matched rather than individually. (iv) Track of yoga practise was assessed through self-report

40	Chanta a et al., 2019, Thailand [44]	Y	(i) Training for 60 minutes per session three times a week for 8 weeks. (ii) The hatha yoga consisted of 10 minutes of warm-up, followed by seated mountain pose (iii) Seated sun pose, boat pose. (iv) Workout for approximately 40 min	(i) IL-2 increased postintervention significantly in the yoga group (*p* < 0.05) but decreased in the control group, indicating that chronic exercise could delay immunosenescence. (ii) IL-6 increased postintervention in both the groups but remains insignificant (*p* > 0.05)	(i) Study is related to respiratory system, which may be important for COVID-19. (ii) Increase in IL-6 contradicts with other yoga intervention

41	Yadav et al., 2018, India [45]	YMP	(i) Active intervention was carried out for 2 hours a day for 14 days, consisting physical postures, pranayama, interactive lecture, and ending with meditative relaxation, and then followed for next 10 weeks at home. (ii) Dietician provided dietary intervention (DI) for both the groups	(i) IL-6 and TBARS levels significantly decreased in the intervention group at two points of analysis. (ii) TNF- *α* and SOD levels changed in groups (both within and between), but were insignificant.	(i) Both groups are homogenous. (ii) Intention to treat analysis could not be carried out. (ii) Home-based interventions have been suffered with noncompliance, which was often difficult to assess

42	Harkess et al., 2016, austrailia [46]	YMP	(i) An hour class of total 16 classes were offered twice weekly for 8 weeks. (ii) Hatha yoga was taught	(i) A moderate correlation (rho =0 .608, *p* < 0.01 between TNF and IL-6 was observed. (ii) The friedman and mixed between–within subjects ANOVA test represented nonsignificant changes in IL-6 or TNF and hsCRP levels, respectively. (iii) Studied in a nonclinical population	(i) The first study to explore DNA methylation. (ii) A pilot study with small sample size and low power of the test

43	Lim SA at al., 2015, Republic of Korea [47]	YMP	(i) 1 day a week for 90 minutes, over 12 weeks (ii) yoga consists of (i) Yoga body poses (asanas for 35 min). (ii) Exercises involving awareness and voluntary regulation of breath (pranayamas for 30 min) (iii) Meditational practices (for 25 min). (iii) Participants attended at least 10 of the total 12 weeks and at least three times at home during the experiment period by watching a 40-minute DVD	(i) The serum TBARS, and IL-12, TNF- *α*, IFN- *γ* reduced significantly in the intervention group and within group comparison respectively. (ii) The serum SOD levels decreased and cortisol levels increased significantly only in the control group.	(i) Many stress related biomarkers were studied. (ii) The sample size was small, and the findings may not be generalized

44	Jorge et al., 2016, Brazil [48]	YMP	(i) 75-min of supervised practices twice a week, for 12 weeks. (ii) Posture included the four typical movement of the vertical column (flexion, extension, lateral bending, and rotation), abdominal fitness practices, and balance exercises.	(i) The salivary cortisol level increased in both the groups, but significant change was observed only in the control group (*p* < 0.001). (ii) The salivary cortisol level decreased in exercise group	(i) At baseline the cortisol level were different. (ii) Uneven attrition was observed in three groups

M = Meditation, P = Pranayama, Y = Yoga, MP = Meditation and pranayama both, YP = Yoga and pranayama both, YM = Yoga and meditation both, and YMP = Combination of yoga, meditation, and pranayama.

**Table 3:** Cochrane risk of bias assessment.

Study name	Random sequence generation	Allocation concealment	Blinding of participants	Blinding of outcome assessment	Incomplete outcome data	Selective reporting	Other bias	Overall risk of bias	Risk of bias rating ^*∗*^
Andres-rodriguez et al. 2019	Low	Low	Some concerns	Low	Low	Low	Some concerns	Low	A1.2
Bower et al. 2015	Low	Low	High	High	Some concerns	Low	High	High	C1.2
Bower et al. 2014	Low	High	High	Some concerns	High	Low	High	High	C1.3
Chanta et al. 2019	Low	High	High	Low	Some concerns	Some concerns	High	High	C1.3
Ganesan et al. 2020	Low	Low	High	Some concerns	Some concerns	Low	Some concerns	Medium	B1.3
Gautam et al. 2019	Low	Low	Some concerns	Low	Low	Low	Some concerns	Low	A1.2
Gautam et al. 2020	Low	Low	Some concerns	Low	Low	Some concerns	Low	Low	A1.2
Nugent NR et al. 2019	Low	Low	High	High	High	Low	High	High	C1.2
Vishwanathan et al. 2020	Low	Low	High	High	High	Low	High	High	C1.2
Gagrani et al. 2018	Low	Low	High	High	Low	Low	Some concerns	High	C1.1
Lipschitz et al. 2013	Low	Low	High	High	Low	Low	High	High	C1.1

Review author's judgments about each risk of bias item across all the included studies. ^*∗*^ Rating was based on the risk of bias. i.e. “low overall bias”—rated as “A,” “medium overall bias” —rated as “B,” and “high overall bias”—rated as “C”. Subsets were further categorized (i.e. C1.0, C1.1.) according to the bias in the individual domain.

**Table 4:** Subgroup analysis.

Parameters	Subgroups	No. of trials	Mean difference [95% CI]	Heterogeneity		Overall Effect	
				Q	I^2^ (%)	Z	*p* Value
IL-6	Yoga-meditation-pranayama	4	−1.44 [−2.33, −0.55]	16.92	82	3.16	0.002
	Meditation only	2	−0.21 [−0.42, 0.00]	0.00	0	1.92	0.05
	Yoga only	2	−0.10 [−0.59, 0.38]	0.04	0	0.42	0.67
	6–10 wk (<1000 min)	3	−1.08 [−2.94, 0.79]	2.12	92	1.13	0.26
	8–12 wk (1000–2000 min)	2	−9.14 [−30.12, 11.84]	7.21	86	0.85	0.39
	6–10 wk (>2000 min)	4	−0.26 [−1.35, 0.82]	107.66	97	0.48	0.63

Cortisol	Meditation only	2	−25.80 [−78.33, 26.73]	63.15	98	0.96	0.34
	6–12 wk (1000–2000 min)	2	−40.75 [−64.13, −17.38]	7.83	87	3.42	0.0006

TNF-*α*	Yoga-meditation-pranayama	4	−3.00 [−6.20, 0.20]	42.08	93	1.84	0.07
	10–12 wk (≤1080 min)	2	−2.46 [−11.40, 6.48]	1.89	47	0.54	0.59
	8–12 wk (3000–4800 min)	3	−3.40 [−4.83, −1.98]	9.53	79	4.68	<0.0001

Interstudy heterogeneity was tested by using the Cochran *Q* statistic (Chi^2^) at a significance level of *p* < 0.10 and quantified by the I^2^ statistic.

**Table 5:** Sensitivity analysis.

Parameters		Mean difference [95% CI]	Heterogeneity		Overall Effect	
			Q	I^2^ (%)	Z	*p*- Value
IL-6	Removing (3) A1.2 studies	−0.42 [−1.34, 0.49]	115.80	96	0.91	0.36
	Removing (1) B1.3 studies	−0.53 −1.28, 0.21]	165.60	96	1.40	0.16
	Removing (3) C1.2 studies	−0.67 [−1.31, −0.04]	15.36	67	2.09	0.04
	Removing (2) C1.3 studies	−0.71 [−1.60, 0.17]	171.86	97	1.58	0.11

TNF-*α*	Removing (2) A1.2 studies	−1.41 [−3.81, 1.00]	48.33	96	1.15	0.25
	Removing (1) B1.3 studies	−2.53 [−4.20, −0.86]	58.10	95	2.96	0.003
	Removing (2) C1.2 studies	−4.19 [−5.29, −3.09]	0.75	0	7.44	<0.00001

Cortisol	Removing (1) B1.3 studies	−25.80 [−78.33, 26.73]	63.15	98	0.96	0.34

Sensitivity analysis, showing progressive effects on pooled mean differences of removing data by trials' risk of bias rating. Interstudy heterogeneity was tested by using the Cochran *Q* statistic (Chi^2^) at a significance level of *p* < 0.10 and quantified by the I^2^ statistic.

**Table 6:** Comparison of the selected biomarkers among COVID-19, other patients, and healthy participants.

Biomarkers	COVID-19 patients (nonRCTs after disease emergence)		Intervention type and dose min (range)	
	Mild/moderate/noncritically ill (compared to control or average)	Severe/critical/ICU/dead	^Ψ^Diseased	Healthy
1. Lymphocytes^†^	↑ ^***γ***^(T cells) [58]	↓ [58,^***σ***^59]	^*∗*^↑ CD4+; YM (840–1200) [33,36]	___
2. IL-6	No change [58] ↓ [63]	↑ [^***σ***^58, ^***σ***^63]	↓ *M* (1890) [18], MP (1260) [53], YP (1200–3000) [23,32], YMP (4800) [40,42]	↓ YP [24], YMP (4800) [45]
3. IL-10	↑ [58]	↑ [58,63]	___	↑ YMP (3240) [37]
4. CRP	↑ [58] ↓ [63]	↑ [^***σ***^58, ^***σ***^60,61,62, ^***σ***^63,^***δ***^64]	↓ YMP (3600–4800) [38,40]	↓ M (1200) [15]
5. IL-1*β*	↑ [63]	↑ [65]	↓ YP (2160) [27]	↓ YMP (960–3240) [26,37]
6. Cortisol	___	↑ [^***δ***^64]	↓ MP (1260) [53], *M* (1890) [18], YP (1440) [31], YMP (1080–1400) [25,56]	↑ YMP (1080) [37]
7. TNF-*α*	↓ [63]	↑ [^***ϕ***^66, ^***σ***^67]	↓ YMP (960–4800) [25,40,42,46], MP (1260) [53], YP (1800–3000) [23,55], YM (980) [35]	↓ YMP (960) [26], YP (2160) [24]

^*∗*^Nonsignificant. ^**†**^Lymphocytes (CD4+, CD8+, B cells, and natural killer cells). ^***γ***^Moderately increase. ^***σ***^Higher compared to that of mild/moderate cases. ^***δ***^Compared to COVID-19 negative ICU patients. ^***ϕ***^Compared to survived COVID-19 patients. ^**Ψ**^The disease participants were diabetes, cancer, RA, HF, and so on, but not COVID-19 patients. ↑=Increase; ↓=decrease; -=no association or study not found.

## Data Availability

Data extracted from the studies, of systematic reviews and meta-analysis, are provided by the corresponding author upon request.

## Additional Points

Highlights of the review. What we knew previously: Some practices of complementary and alternative medicine (CAM), traditional Chinese medicine (TCM), and herbal medicine possibly have positive effects on COVID-19 patients. Biomarkers (around 25) are found to be favourably changed by yoga, meditation, and pranayama (breathing exercises) (YMP) among different clinical (other than COVID-19) and healthy participants. Different biomarkers, leading to cytokine storm, are affected in COVID-19. What this review added on: YMP interventions can change the levels of biomarkers favouring health status of healthy, clinical, and the pregnant women with nine, fourteen, and two biomarkers, respectively, altogether 25, which may have possible implications in COVID-19. Clearly, combined action of YMP with specific durations might be effective in reducing IL-6, cortisol, and TNF-*α* among the COVID-19 patients.

## Supplementary Materials

Supplementary Materials S1_Search Strategy with Keywords and MeSH.
