# Supplementary material for: Yoga, Meditation, Breathing Exercises, and Inflammatory Biomarkers with Possible Implications in COVID-19: A Systematic Review and Meta-Analysis of Randomized Controlled Trials
Source: Evid Based Complement Alternat Med. 2022 Oct 7;2022:3523432. doi: 10.1155/2022/3523432 (PMC9568285; doi:10.1155/2022/3523432)
Supplement: Supplementary Materials — S1_Search Strategy with Keywords and MeSH. [file 3523432.f1.docx]

Table 1. Search results in three databases (Cochrane, Pubmed and Google scholar)

| **SN (ID)** | **MeSH/Key words** | **Search strategy** | **Hits** | **Remarks** | |
| --- | --- | --- | --- | --- | --- |
| 1. **Cochrane search strategy: date** 08/11/2021 06:36:35 | | | | | |
| #1 | MeSH descriptor: [Yoga] explode all trees | | 748 | |  |
| #2 | (yogasana or asana or yoga or kriya or mudra):ti,ab,kw | | 17 | |  |
| #3 | #1 or #2 | | 758 | |  |
| #4 | MeSH descriptor: [Meditation] explode all trees | | 671 | |  |
| #5 | (dhyana or mudra or shunya or chanting or chant or singing or chants):ti,ab,kw | | 2 | |  |
| #6 | #4 or #5 | | 673 | |  |
| #7 | MeSH descriptor: [Breathing Exercises] explode all trees | | 953 | |  |
| #8 | (pranayama or bhastrika or kapalbhati):ti,ab,kw | | 2 | |  |
| #9 | #7 or #8 | | 955 | |  |
| #10 | #3 or #6 or #9 | | 2179 | |  |
| #11 | MeSH descriptor: [Biomarkers] explode all trees | | 21586 | |  |
| #12 | (immunity or interleukin* or interferon* or "c-reactive" or immunolog*):ti,ab,kw | | 595 | |  |
| #13 | ("immunologic marker" or "antigens cd") | | 7 | |  |
| #14 | #11 or #12 or #13 | | 22105 | |  |
| #15 | #10 AND #14 | | 46 | |  |
| #16 | Filters; date Between Oct 31, 2011 and Oct 30, 2021, in Trials (including the word variations have been searched) | | 40 | |  |
| 1. **PubMed search strategy** | | | | | |
|  | (("Yoga"[MeSH Terms] OR ("yogasana"[Title/Abstract] OR "asana"[Title/Abstract] OR "Yoga"[Title/Abstract] OR "kriya"[Title/Abstract] OR "mudra"[Title/Abstract]) OR ("Meditation"[MeSH Terms] OR ("dhyana"[All Fields] OR ("chanting"[All Fields] OR "singing"[MeSH Terms] OR "singing"[All Fields] OR "chant"[All Fields] OR "chants"[All Fields]))) OR ("Breathing Exercises"[MeSH Terms] OR ("pranayama"[Title/Abstract] OR "bhastrika"[Title/Abstract]))) AND ("Biomarkers"[MeSH Terms] OR ("immunity"[Title/Abstract] OR "interleukin*"[Title/Abstract] OR "interferon*"[Title/Abstract] OR "c-reactive protein"[Title/Abstract] OR "immunologic marker"[All Fields] OR "antigens cd"[All Fields]))) AND ((clinicaltrial[Filter]) AND (fft[Filter]) AND (humans[Filter]) AND (2011/10/31:2021/10/31[pdat]) AND (english[Filter])) | | 70 | |  |
| 1. **Google scholar search strategy** | | |  | |  |
| Google scholar search for “yoga” | (Yoga (all of the words); controlled trial (exact word); biomarkers asana yogasana Iyengar hatha hata hot astanga pranayama breathing kriya mudra immunity immunoglobulin respiratory (with at least one of the word) | Search Details: allintitle: yoga biomarkers OR asana OR yogasana OR iyengar OR hatha OR hata OR hot OR astanga OR pranayama OR breathing OR kriya OR mudra OR immunity OR immunoglobulin OR respiratory "controlled trial" | 27 | | [Filters; only English; title of the articles; dated 2011-2021] |
|  | (Yoga or prospective (all of the words); biomarkers asana yogasana iyengar hatha hata hot astanga pranayama breathing kriya mudra immun immunoglobulin respiratory interleukin interferon "c reactive" telomere (with at least one of the word) | allintitle: yoga prospective biomarkers OR asana OR yogasana OR iyengar OR hatha OR hata OR hot OR astanga OR pranayama OR breathing OR kriya OR mudra OR immun OR immunoglobulin OR respiratory OR interleukin OR interferon OR "c reactive" OR telomere | 3 | |  |
|  | (Yoga or cohort (all of the words); biomarkers asana yogasana iyengar hatha hata hot astanga pranayama breathing kriya mudra immun immunoglobulin respiratory interleukin interferon "c reactive" telomere (with at least one of the word) | allintitle: yoga cohort biomarkers OR asana OR yogasana OR iyengar OR hatha OR hata OR hot OR astanga OR pranayama OR breathing OR kriya OR mudra OR immun OR immunoglobulin OR respiratory OR interleukin OR interferon OR "c reactive" OR telomere | 1 | | [Filters; only English; title of the articles; dated 2011-2021] |
|  | (Yoga or longitudinal (all of the words); biomarkers asana yogasana iyengar hatha hata hot astanga pranayama breathing kriya mudra immun immunoglobulin respiratory interleukin interferon "c reactive" telomere (with at least one of the words) | allintitle: yoga longitudinal biomarkers OR asana OR yogasana OR iyengar OR hatha OR hata OR hot OR astanga OR pranayama OR breathing OR kriya OR mudra OR immun OR immunoglobulin OR respiratory OR interleukin OR interferon OR "c reactive" OR telomere | 1 | |  |
| Google scholar search for “meditation” | Meditation (all of the words); controlled trial (exact word); biomarkers dhyana mudra immun immunoglobulin respiratory interleukin interferon "c reactive" OR telomere (with at least one of the word) | allintitle: meditation biomarkers OR dhyana OR mudra OR immun OR immunoglobulin OR respiratory OR interleukin OR interferon OR "c reactive" OR telomere "controlled trial" | 8 | | [Filters; only English; title of the articles; dated 2011-2021] |
|  | Meditation or cohort (all of the words); biomarkers dhyana mudra immun immunoglobulin respiratory interleukin interferon "c reactive" OR telomere (with at least one of the word) | allintitle: meditation cohort biomarkers OR dhyana OR mudra OR immun OR immunoglobulin OR respiratory OR interleukin OR interferon OR "c reactive" OR telomere | 0 | |  |
|  | Meditation or longitudinal (all of the words); biomarkers dhyana mudra immun immunoglobulin respiratory interleukin interferon "c reactive" OR telomere (with at least one of the word) | allintitle: meditation longitudinal biomarkers OR dhyana OR mudra OR immun OR immunoglobulin OR respiratory OR interleukin OR interferon OR "c reactive" OR telomere | 0 | |  |
|  | Meditation or prospective (all of the words); biomarkers dhyana mudra immun immunoglobulin respiratory interleukin interferon "c reactive" OR telomere (with at least one of the word) | allintitle: meditation prospective biomarkers OR dhyana OR mudra OR immun OR immunoglobulin OR respiratory OR interleukin OR interferon OR "c reactive" OR telomere | 0 | |  |
| Google scholar search for “pranayama” | Breathing exercises (all of the words); controlled trial (exact word); biomarkers pranayama immun immunoglobulin respiratory interleukin interferon "c reactive" OR telomere (with at least one of the word) | allintitle: Breathing exercises biomarkers OR pranayama OR immun OR immunoglobulin OR respiratory OR interleukin OR interferon OR "c reactive" OR telomere "controlled trial" | 1 | | [Filters; only English; title of the articles; dated 2011-2021] |
|  | Breathing exercises cohort (all of the words); biomarkers pranayama immun immunoglobulin respiratory interleukin interferon "c reactive" OR telomere (with at least one of the word) | allintitle: Breathing exercises cohort biomarkers OR pranayama OR immun OR immunoglobulin OR respiratory OR interleukin OR interferon OR "c reactive" OR telomere | 0 | |  |
|  | Breathing exercises longitudinal (all of the words); biomarkers pranayama immun immunoglobulin respiratory interleukin interferon "c reactive" OR telomere (with at least one of the word) | allintitle: breathing exercises longitudinal biomarkers OR pranayama OR immun OR immunoglobulin OR respiratory OR interleukin OR interferon OR "c reactive" OR telomere | 0 | |  |
|  | Total (Google scholar) |  | 41(-2) | |  |
| **All Total (3 databases)** | |  | **151(-2)** | | **=149** |
